# Endoscopic biliary drainage outperforms conventional external drainage in pediatric choledochal cyst with severe cholangitis: a retrospective cohort study

**DOI:** 10.3389/fped.2025.1648834

**Published:** 2025-10-09

**Authors:** Hongxi Guo, Juan Luo, Jingjing Chen, Jun Yang, Hongqiang Bian, Hu Yang, Xufei Duan, Xin Wang

**Affiliations:** ^1^ Department of General Surgery, Wuhan Children’s Hospital, Tongji Medical College, Huazhong University of Science & Technology, Wuhan, China; ^2^ Department of Endocrinology and Metabolism, Wuhan Children’s Hospital, Tongji Medical College, Huazhong University of Science & Technology, Wuhan, China

**Keywords:** choledochal cyst, severe cholangitis, endoscopic biliary drainage, conventional external drainage, children

## Abstract

**Background:**

Choledochal cyst (CC) is a common biliary malformation in children and is often associated with severe cholangitis. While endoscopic biliary drainage (EBD) is well established in adults, its efficacy and safety in children remain unclear. This study aimed to compare the effectiveness and safety of EBD with conventional external drainage (CED) in children with CC complicated by severe cholangitis.

**Methods:**

Clinical data from children with CC complicated by severe cholangitis who were treated at Wuhan Children's Hospital between January 1, 2013, and January 31, 2025, were retrospectively analyzed. Patients were divided into CED and EBD groups based on the drainage method. Various clinical outcomes were compared between the two groups, including operative time, intraoperative bleeding, postoperative hospitalization duration, pain scores, complication rates, time to radical surgery, conversion to open surgery, and laboratory indices.

**Results:**

Fifty-nine children (CED: *n* = 31; EBD: *n* = 28) were included (12 males and 47 females, with a mean age of 3.21 ± 2.17 years). Compared with CED, the EBD group demonstrated significantly better outcomes, including a shorter operative time (*P* < 0.001), less blood loss (*P* < 0.001), reduced length of stay (3.2 vs. 6.5 days, *P* < 0.001), lower pain scores (2.1 vs. 5.8, *P* < 0.001), fewer complications (3.57% vs. 25.81%, *P* = 0.044), shorter radical surgery interval (14 vs. 28 days, *P* = 0.002), and lower laparotomy conversion rate (3.57% vs. 29.03%, *P* = 0.024). Both groups demonstrated statistically significant differences in postoperative 24 h reductions of total bilirubin and transaminase levels (*P* < 0.05).

**Conclusions:**

In children with CC complicated by severe cholangitis, EBD provides significant advantages over CED. It is less invasive, which leads to faster recovery times and lower complication rates, making it the preferred transitional treatment before definitive surgery.

## Introduction

Choledochal cyst (CC), also known as congenital biliary dilatation, is a congenital malformation characterized by cystic or spindle-shaped dilatation of the bile ducts. The incidence of CC is significantly higher in Asian children than in Western populations, with an estimated occurrence of approximately 1:100,000–150,000 live births (1–3). Additionally, the condition is more common in females, with a male-to-female ratio of 1:3 to 1:4. More than 60% of cases are diagnosed before the age of 10 years (4). If left untreated, CC can lead to severe complications, including cholangitis, pancreatitis, cystic perforation, and even bile duct cancer (5, 6). Acute cholangitis is a relatively common complication of CC, presenting symptoms such as fever, jaundice, abdominal pain, and a systemic inflammatory response. The symptoms are often mild and can be improved with conservative treatment. However, some children may present with severe symptoms at onset or experience symptoms progression despite conservative management. For these patients, urgent biliary decompression is critical to prevent severe sepsis or bile duct perforation (7–9).

The primary treatment for CC complicated by severe cholangitis involves biliary drainage. Conventional external biliary drainage (CED), which includes T-tube drainage, percutaneous cystocentesis, and cholecystostomy, is commonly used. However, these techniques are associated with significant drawbacks, including incisional pain, scarring, and complication rates of up to 25%, with risks such as incisional infection and intestinal adhesions (7, 10). While radical surgery, including Roux-en-Y hepaticojejunostomy, is the definitive treatment for CC, acute inflammation often necessitates temporary biliary drainage as a transitional measure (11, 12). Cyst excision and biliary reconstruction can be particularly challenging due to the risk of intraoperative bleeding and postoperative complications, including difficulties with infection control and anastomotic failure.

Endoscopic retrograde cholangiopancreatography (ERCP) is widely used as a diagnostic and therapeutic tool for pancreaticobiliary disorders in adults and has also proven effective and safe in pediatric patients (8, 13–17). While endoscopic biliary drainage (EBD) is well-established in adults, its use in pediatrics remains limited due to technical challenges, equipment constraints, and a lack of specialized expertise (17). Moreover, there is no study comparing the efficacy of EBD and CED in the treatment of children with CC complicated by severe cholangitis.

In this study, we used endoscopic nasobiliary drainage or stent placement between the dilated common bile duct and the duodenum to facilitate bile drainage in children with CC complicated by severe cholangitis. The study aimed to systematically compare the clinical efficacy and safety of EBD with CED in treating pediatric CC complicated by severe cholangitis and to provide an evidence-based foundation for optimizing treatment protocols.

## Materials and methods

### Patients

This study was approved by the Ethics Committee of Wuhan Children's Hospital (IRB No. 2024R032-E01), with all participants’ personal information kept confidential. Written informed consent was obtained from all participants’ parents or legal guardians. All operations in the study strictly adhered to the relevant guidelines and regulations.

This retrospective, single-center study reviewed the clinical data of all patients diagnosed with CC who received treatment at the Department of General Surgery, Wuhan Children's Hospital, between January 1, 2013, and January 31, 2025. Wuhan Childre's Hospital is a leading tertiary pediatric center that serves patients from across China and is one of the few hospitals in the country offering ERCP services for pediatric patients.

The inclusion criteria were as follows: (1) Age <18 years; (2) diagnosis of CC with severe cholangitis, confirmed by ultrasound, magnetic resonance imaging, or computed tomography. Severe cholangitis in this study was defined by the presence of fever (temperature >38.5 °C), abdominal pain, jaundice [total bilirubin (TB) > twice the upper limit of normal], tachycardia, elevated respiratory rate, increased or decreased white blood cell count (WBC), elevated C-reactive protein, and abnormal liver function [alanine aminotransferase [ALT] and aspartate aminotransferase [AST] > twice the upper limit of normal] (18). (3) Patients who did not indicate improvement within 72 h of conservative treatment with fasting, antibiotics, and antispasmodics (no improvement in fever and leukocytosis following intravenous antibiotics and metronidazole) were included in the study. (4) All patients ultimately underwent choledochal cystectomy and biliary tract reconstruction at our hospital.

The exclusion criteria were as follows: (1) Presence of biliary perforation, septic shock, or unstable vital signs and (2) missing clinical information.

**Figure 1:**
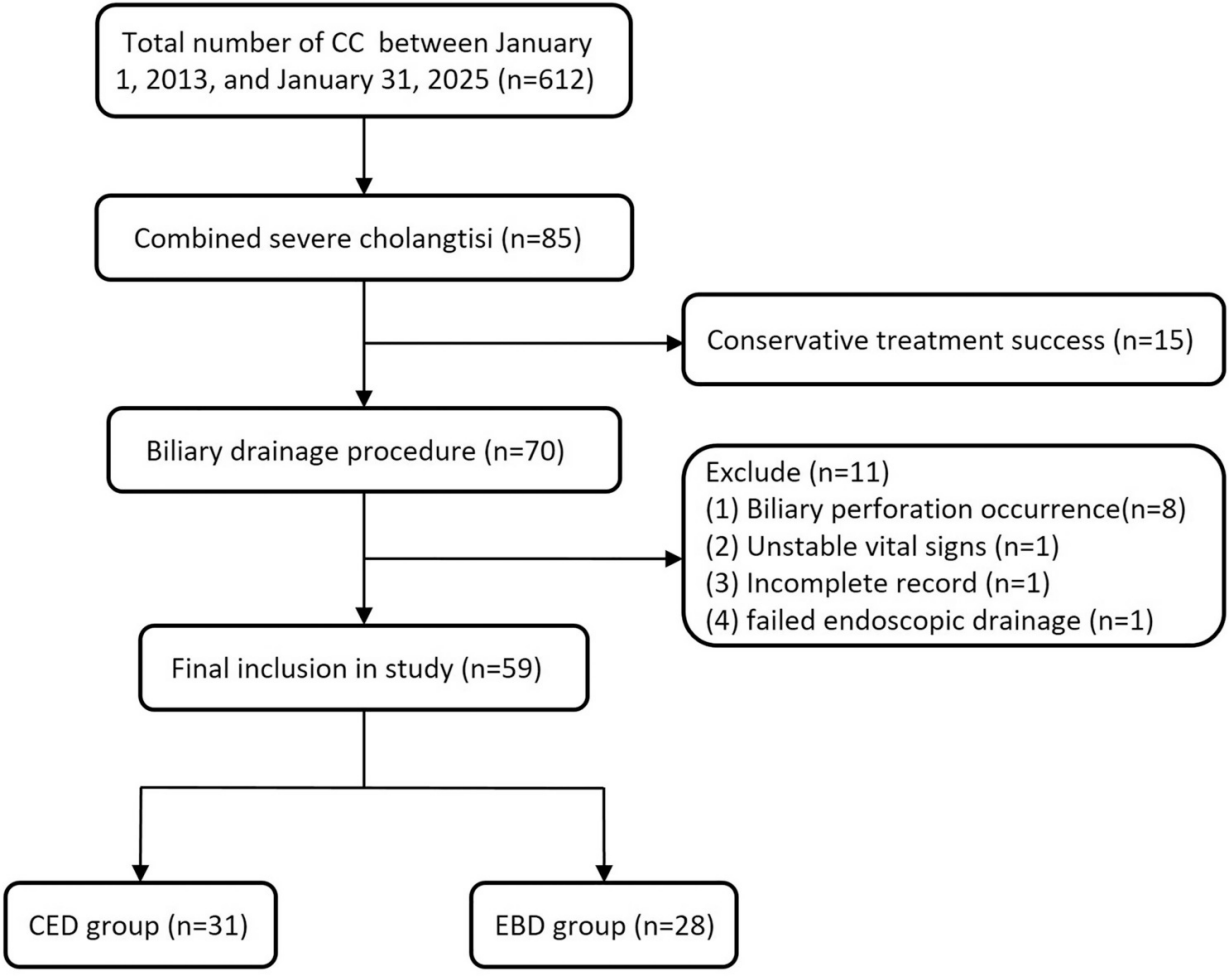
Flowchart of the study.

During the study period, a total of 612 children with CC were admitted to the Department of General Surgery at Wuhan Children's Hospital. Among them, 85 cases were complicated with severe cholangitis, and 70 patients ultimately required biliary drainage due to unsuccessful conservative therapy. One case of CC with severe cholangitis failed drainage due to a large choledochal cyst. Although a guidewire was successfully inserted into the bile ducts, placing a drainage accessory (nasobiliary tube or stent) was impossible. This patient subsequently underwent laparoscopic cyst drainage and was excluded from the study (Figure 1).

### Surgical procedure

The allocation of patients to either the conventional external drainage (CED) or endoscopic biliary drainage (EBD) group was guided by two principal criteria to ensure both clinical appropriateness and ethical integrity. First, physiological suitability was rigorously evaluated. Given that EBD involves endoscopic manipulation of the biliary system—a procedure demanding considerable technical expertise and compatible anatomical development—children under one year of age or with a body weight below 10 kg were considered suboptimal candidates for EBD. In such cases, CED was recommended as the primary drainage intervention. Second, informed consent was obtained from all legal guardians following a comprehensive discussion led by the attending surgeon. This consultation detailed the potential risks (e.g., pancreatitis or hyperamylasemia associated with EBD; incision infection, drain dislodgement, or adhesion formation related to CED), anticipated benefits (e.g., minimal invasiveness and reduced pain with EBD; immediate decompression efficacy with CED), and expected recovery trajectories associated with each procedure. The final treatment decision was made collaboratively with the guardians, and written informed consent was secured prior to intervention.

All procedures were performed under general anesthesia following a standardized perioperative protocol. Anesthesia and analgesia were administered by two experienced pediatric anesthesiologists, with consistent use of intraoperative opioids and postoperative pain management strategies across both groups in accordance with institutional guidelines. It is noteworthy that there were no significant differences in baseline demographic, clinical, or laboratory characteristics between the two groups (Table 1), supporting the appropriateness of the allocation protocol.

**Table 1:** Comparison of preoperative baseline characteristics between CED and EBD groups.

Characteristics	CED group (n = 31)	EBD group (n = 28)	*P*-value
Age (years)	2.94 ± 1.96	3.51 ± 2.38	0.318
M/F	7/24	5/23	0.653
Other clinical manifestations			
Abdominal distension, *n* (%)	5 (16.13)	3 (10.71)	0.821
Vomiting or/and nausea, *n* (%)	17 (54.84)	14 (45.16)	0.710
Itchy skin, *n* (%)	7 (22.58)	6 (21.43)	0.915
Laboratory tests			
WBC (1 × 10^9^/L)	15.22 ± 3.26	16.33 ± 3.10	0.185
TB (μmol/L)	47.60 (40.20, 64.25)	47.60 (40.20, 64.25)	0.544
DBIL (μmol/L)	35.00 (30.20, 46.90)	34.75 (30.88, 56.25)	0.693
ALT (U/L)	106.00 (95.00, 155.00)	159.50 (92.00, 312.75)	0.346
AST (U/L)	115.00 (97.50, 174.00)	124.00 (91.00, 232.50)	0.820
SAMY (U/L)	111.00 (71.50, 254.50)	134.50 (92.00, 268.00)	0.471
Cyst type			
Ⅰ/IV	26/5	22/6	0.602

In the second and third columns, metric data are presented as the mean ± standard deviation or median (1st quantile, 3rd quantile), and count data are expressed as *n* (%).

WBC, white blood cell; TB, total bilirubin; DBIL, direct bilirubin; ALT, alanine aminotransferase; AST, aspartate aminotransferase; SAMY, serum amylase.

### CED group

#### Laparoscopic external cholecystic drainage

**Figure 2:**
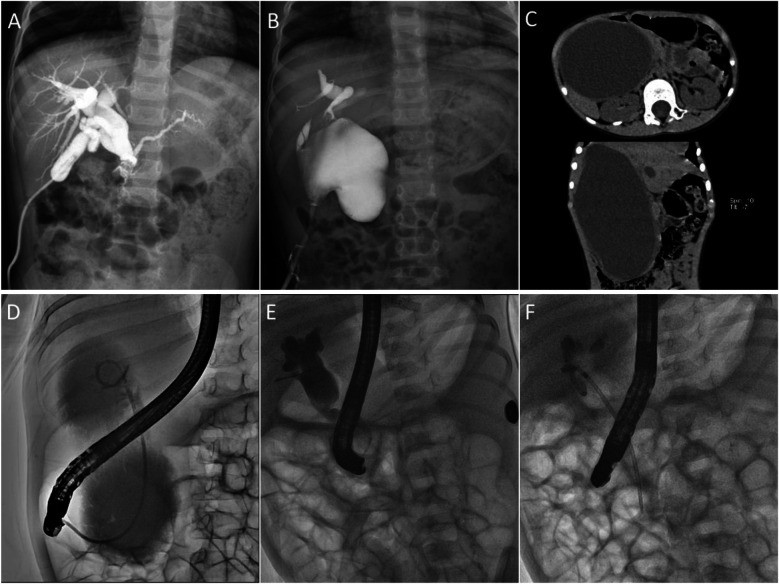
Imaging findings of different drainage modalities. **(A)** Laparoscopic external drainage of the gallbladder; **(B)** Laparoscopic external drainage of the bile duct; **(C,D)** A 5-year and 7-month-old child with a substantial common bile duct associated with severe cholangitis underwent EBD. **(C)** CT image indicated that the cysts were huge, and the size of the cysts was about 16 × 10 cm. **(D)** Under fluoroscopic guidance during ERCP, a 7-French nasobiliary catheter was selectively positioned within the cyst to establish continuous biliary decompression, and the cysts shrunk to 4 × 3 cm three weeks after the operation. **(E,F)** Under fluoroscopic ERCP guidance, a 7 Fr–7 cm pancreatic duct stent was placed reversely for internal biliary drainage.

A trocar was inserted through an umbilical incision to create an observation port. A 1 cm skin incision was then made at the surface projection of the body of the gallbladder base. Vascular forceps were used to bluntly dissect and expand the incision into the abdominal cavity, allowing the gallbladder to be pulled through the abdominal wall incision. A pre-positioned retrieval bag was used, and the gallbladder was incised. An 8–12 F silicone dual-lumen catheter was inserted, and the purse-string suture was tightened upon observing bile outflow. Saline was injected into the balloon to prevent prolapse. After confirming the absence of the choledochal duct stenosis, the gallbladder was returned to the abdominal cavity. The procedure concluded with repeated bile suction, saline water injection, and thorough irrigation (Figure 2A).

#### Laparoscopic external drainage of the cyst

A 0.5 cm incision was made on the anterior cyst wall using laparoscopy, and a T-tube was inserted into the cyst. A T-tube was then fixed using purse-string sutures (Figure 2B).

#### Percutaneous cyst puncture drainage

For children with large cysts, percutaneous drainage was performed under local anesthesia and ultrasound guidance. Using the Seldinger technique, a pigtail catheter is inserted into the cystic cavity for drainage.

### EBD group

The child was placed in a prone position under general anesthesia. A duodenoscope (JF-260 V, 11.3, and 3.7 mm operative channel or TJF-260 V, 11.3, and 4.2 mm operative channel; Olympus, Tokyo, Japan) was advanced to the duodenal papilla. Using a guidewire-assisted technique, a papillotomy knife (Anrei, Hangzhou, China) was introduced, followed by selective bile duct intubation and cholangiography to confirm the biliary anatomy. A guidewire (Anrei, Hangzhou, China) was then inserted into the bile duct, and either a nasobiliary drainage tube (Cook, Limerick, Ireland) or a stent was advanced along the guidewire into the cyst for bile drainage (Figures 2D–F). Due to the unavailability of pediatric biliary stents, pancreatic duct stents (7Fr–7 cm; Cook, Limerick, Ireland) were used for ERCP-guided endobiliary drainage (Figure 2F). All equipment used was originally designed for adult ERCP procedures.

To prevent tube dislodgement in pediatric patients, particularly in young or irritable children, the nasobiliary tube was securely affixed to the cheek and forehead with adhesive tape. A protective nasal bridge pad was applied to minimize cutaneous irritation. Comprehensive caregiver education was provided regarding tube management and traction prevention. Select cases utilized a lightweight coiled tube configuration to mitigate tension. These protocols ensured effective drainage maintenance throughout the preoperative period.

### Data collection

The following data were collected for all children: (1) Intraoperative data: Operative time, intraoperative bleeding, and drainage method used; (2) Postoperative outcomes: Postoperative hospitalization duration, time to first feeding, FLACC pain scores (19) at 24 and 48 h postoperatively, and postoperative complications (blocked or dislodged drainage tubes, incisional infections, intestinal adhesions, pancreatitis, gastrointestinal perforation, and hemorrhage). Additionally, the interval between the initial external drainage and subsequent radical surgery was recorded, along with whether laparoscopic or robotic radical surgery was performed with or without an intermediate open surgery; (3) Laboratory tests: Preoperative tests (based on the first test following admission) and postoperative tests at 24 h, including WBC, TB, direct bilirubin (DBIL), ALT, AST, gamma-glutamyl transpeptidase (GGT), and blood and urine amylase levels.

### Statistical analysis

SPSS version 25.0 for Windows (IBM Corp., Armonk, NY, USA) was used for all statistical analyses. Data were tested for normality using the Shapiro–Wilk test. Normally distributed data are expressed as means and standard deviations and compared using the *t*-test. Data with skewed distributions were expressed as median (1st quantile, 3rd quantile), and group comparisons were made using the Mann–Whitney *U* test. Categorical data are expressed as counts and percentages, with comparisons between groups using the Pearson chi-square or Fisher's exact test. A *P* < 0.05 was considered statistically significant.

## Results

### Patient baseline characteristics

After applying the inclusionb and exclusion criteria, 59 children were ultimately included in the study (12 males and 47 females), with an age range of 0.6 months to 10.7 years and a mean age of 3.21 ± 2.17 years. According to the drainage method, the children were divided into two groups: The CED group (*n* = 31) and the EBD group (*n* = 28).

In the CED group, there were 31 patients (7 males and 24 females) with a mean age of 2.94 ± 1.96 years. The procedures included 23 cases of laparoscopic external cholecystic drainage, 6 laparoscopic external cystic drainage cases, and 2 percutaneous cystic puncture drainage cases. According to Todani's classification (20), there were 26 type I and 5type IV cases. In the EBD group, there were 28 patients (5 males and 23 females) with a mean age of 3.51 ± 2.38 years. This group included two cases of biliary stent drainage (7Fr–7 cm pancreatic duct stent used as an alternative) and 26 cases of nasobiliary drainage. Regarding the Todani classification, there were 22 type I and 6 cases of type IV.

There were statistically non-significant differences in age, gender, or classification between the two groups (*P* > 0.05). All patients exhibited abdominal pain, jaundice, and fever, along with other clinical symptoms such as abdominal distension, vomiting, and skin itching. The two groups exhibited statistically non-significant differences in these clinical manifestations (*P* > 0.05). Laboratory tests, including leukocyte count, DBIL, aminotransferases, and amylase levels, also indicated non-significant differences between the groups (*P* > 0.05). Baseline characteristics and clinical manifestations are summarized in Table 1.

### Comparison of perioperative outcomes

The operative time and intraoperative bleeding were significantly shorter in the EBD group compared to the CED group. The Length of stay was also shorter in the EBD group. However, the two groups had statistically non-significant differences regarding time to initial feeding (*P* = 0.305). In the CED group, 61% of children required an analgesic medication, whereas only one case (3.57%) in the EBD group needed analgesic medication. The FLACC pain scores were significantly higher in the CED group compared to the EBD group at 24 and 48 h postoperatively, with statistically significant differences between the groups (*P* < 0.05) (Table 2).

**Table 2:** Comparison of intraoperative and postoperative conditions, FLACC scores at different postoperative time points, and radical surgery between CED and EBD groups.

Characteristics	CED group (*n* = 31)	EBD group (*n* = 28)	*P*-value
Operative time (min)	65.00 (55.00, 75.00)	29.00 (25.00, 32.50)	<.001
Intraoperative bleeding (ml)	5.00 (5.00, 10.00)	0.00 (0.00, 0.10)	<.001
length of stay (days)	65.00 (55.00, 75.00)	29.00 (25.00, 32.50)	<.001
Time to first oral intake (days)	2.00 (2.00, 3.00)	3.00 (2.00, 3.00)	0.305
Postoperative complications (*n*%)	8 (25.81)	1 (3.57)	0.044
FLACC score			
24 h after surgery	4.00 (3.00, 4.00)	2.00 (1.00, 2.00)	<.001
48 h after surgery	1.00 (1.00, 2.00)	1.00 (0.00, 1.00)	<.001
the interval for definite surgery (days)	21.00 (14.00, 30.00)	14.00 (10.00, 19.50)	0.002
Conversion to open surgery (*n*%)	9 (29.03)	1 (3.57)	0.024

In the second and third columns, metric data are presented as the mean ± standard deviation or median (1st quantile, 3rd quantile), and count data are expressed as *n* (%); FLACC score, Each of the five categories (F) Face; (L) Legs; (A) Activity; (C) Cry; (C) Consolability is scored from 0 to 2, which results in a total score between zero and ten.

### Postoperative laboratory findings

The levels of TB, ALT, and AST decreased to varying degrees 24 h postoperatively in CED and EBD groups compared to preoperative levels, and the differences between the two groups were statistically significant (*P* < 0.05 for all, Table 3).

**Table 3:** Comparison of 24 h postoperative laboratory findings between CED and EBD groups.

Characteristics	CED group (*n* = 31)	EBD group (*n* = 28)	*P*-value
WBC (1 × 10^9^/L)	7.05 ± 2.52	7.98 ± 2.37	0.154
TB (μmol/L)	16.20 (11.10, 19.85)	8.40 (6.08, 15.30)	0.019
DBIL μmol/L)	7.10 (3.90, 11.25)	6.80 (2.75, 9.27)	0.569
ALT (U/L)	49.00 (42.50, 80.00)	36.00 (19.50, 56.75)	0.009
AST (U/L)	49.00 (41.00, 79.00)	37.00 (23.00, 48.75)	0.007
GGT (U/L)	69.00 (74.50, 460.50)	195.50 (138.25, 376.00)	0.523
SAMY (U/L)	67.00 (46.50, 85.50)	66.00 (47.50, 110.25)	0.915
UAMY (U/L)	135.00 (56.00, 227.00)	142.50 (69.25, 313.50)	0.268

In the second and third columns, metric data are presented as the mean ± standard deviation or median (1st quantile, 3rd quantile), and count data are expressed as *n* (%).

WBC, white blood cell; TB, total bilirubin; DBIL, direct bilirubin; ALT, alanine aminotransferase; AST, aspartate aminotransferase; GGT, gamma-glutamyl transpeptidase; SAMY, serum amylase; UAMY, urinary amylase.

### Postoperative complications

Home care was provided for children in both the CED group and those with nasobiliary drainage in the EBD group to prevent drain dislodgement or incision infection. In the CED group, there were three cases of drain detachment (9.68%: Two cases following laparoscopic cholecystic drainage and one case after laparoscopic cystic drainage). One case of drain obstruction (3.23%) occurred following laparoscopic cholecystic drainage, while two cases of incision infection (6.45%), one case of intestinal obstruction (3.23%), and one case of bowel adhesion (3.23%) were reported. One of the three cases of drain detachment was successfully reinserted through the original sinus tract. In comparison, in two cases, reinsertion failed, and the patients underwent radical bile-intestinal anastomosis earlier than scheduled. In one case of drain obstruction, cholangiography revealed a tortuous gallbladder duct blocked by stones. A nasobiliary tube was successfully inserted endoscopically into the cyst to restore drainage. Children with intestinal obstruction and intestinal adhesion were managed conservatively with fasting, gastrointestinal decompression, anti-infective therapy, and other conservative treatments. No cases of drain dislodgement or obstruction occurred in the EBD group. One child in the EBD group (3.57%) developed postoperative hyperamylasemia, which resolved after three days of fasting, enzyme inhibition, and nutritional support. The overall complication rate was significantly higher in the CED group (25.81%) compared to the EBD group (3.57%), with a statistically significant difference (*P* < 0.001) (Table 2).

### Subsequent radical surgery

A total of 59 patients ultimately underwent radical surgery, including 19 robotic procedures, 29 laparoscopic procedures, and 11 open surgeries. In laparoscopic external gallbladder drainage cases and subsequent radical surgery following percutaneous cystocentesis and drainage, adhesions were primarily observed between the gallbladder base and the abdominal wall, with minimal adhesions and less inflammation around the common bile duct. However, in final surgeries following laparoscopic biliary drainage, adhesions were visible around the choledochal fistula, intestines, and greater omentum. The subhepatic margin covered the anterior wall of the choledochal fistula, while the hepaticoduodenal ligament appeared edematous and thickened, with clear signs of inflammation in the fistula wall. Although no obvious adhesions were found in the abdominal cavity of patients in the ERCP group, varying degrees of edema were observed in the distal bile duct wall during subsequent radical surgery. Analysis revealed that the interval between radical surgeries was shorter in the EBD group than in the CED group, and the rate of conversion to open surgery was lower in the EBD group. These differences were statistically significant (*P* < 0.05) (Table 2).

## Discussion

Acute biliary tract infection is one of the most common complications of CC. In children with CC and severe biliary infections, external drainage is typically indicated when the condition cannot be controlled with a short period of conservative treatment (7–9, 16, 21, 22). Emergency external biliary drainage is recommended as a Phase I intervention, followed by definitive Phase II surgery. Traditional extracorporeal drainage methods, including open or laparoscopic biliary T-tube drainage, gallbladder drainage, and percutaneous cystocentesis drainage, are commonly used after the acute phase has subsided (9, 21, 22). These techniques usually provide immediate symptomatic relief and help reduce biliary inflammation. However, they have significant drawbacks, including painful incisions and the potential development of infections and adhesions in the surrounding tissues, which can increase the difficulty and risk of subsequent radical surgery (22).

The EBD overcomes many of the disadvantages associated with traditional external drainage. The ERCP with nasobiliary or stent placement drainage has been widely used to manage adult pancreaticobiliary diseases. However, using ERCP in children remains limited, primarily due to the lack of specialized pediatric endoscopist equipment for children and the limited experience of pediatric endoscopists (17). Additional challenges include the relatively low prevalence of conditions requiring ERCP in childhood and the perception that the procedure is technically difficult in children (8). Recently, advancements in pediatric duodenoscopy techniques have led to increased research on using ERCP for choledochal cysts and severe cholangitis in children. Tsuchiya et al. (8, 15) concluded that endoscopic stenting is an effective treatment for children with persistent or worsening symptoms of CC. We have successfully applied ERCP for early-stage choledocholithiasis and chronic pancreatitis in children and are gaining additional experience (23). In this study, we performed ERCP with stenting and nasobiliary drainage in children with severe cholangitis caused by CC, achieving favorable clinical outcomes consistent with Sun et al. findings (16).

In this study, we systematically compared the clinical efficacy and safety of EBD with CED in children with CC complicated by severe cholangitis through a retrospective cohort analysis. The results indicated that EBD outperformed CED regarding surgical efficiency (operative time, intraoperative bleeding), postoperative recovery (length of stay, pain scores), and complication control. These findings confirm the feasibility of using endoscopic techniques in pediatric patients with biliary disorders and provide crucial evidence for optimizing transitional treatment strategies for severe cholangitis in CC.

In this retrospective cohort, the operative time in the EBD group (median 29 min) was significantly shorter compared to the CED group (median 65 min), and intraoperative bleeding was nearly negligible (0 vs. 5 ml), a finding that aligns with recent trends in the development of endoscopic techniques in pediatric patients. Studies in adults have indicated that ERCP-guided biliary drainage avoids open surgery and trauma to the abdominal cavity or bile ducts by utilizing the natural lumen, significantly reducing operative time and minimizing intraoperative bleeding risk (1). However, the application of EBD in pediatric patients has been limited due to difficulties, including smaller anatomical structures, lack of specialized equipment, and insufficient experience (17). This study successfully achieved efficient biliary drainage in children using adult-sized ERCP equipment combined with standardized operating procedures.

The minimally invasive nature of EBD also had a direct impact on the postoperative recovery process. The EBD group exhibited a significantly shorter postoperative hospital stay than the CED group (*P* < 0.001). Additionally, the FLACC pain score in the EBD group was lower (median 2) compared to the CED group (median 4) at 24 h postoperatively, and the CED group exhibited a longer hospital stay (median 12 days). This difference can be attributed to the invasive nature of CED surgery, whether open or laparoscopic, which causes greater tissue damage and triggers a stronger inflammatory response. In contrast, EBD is performed through the natural lumen of the endoscope, avoiding abdominal wall incisions and intra-abdominal instrumentation. Consequently, it significantly reduces postoperative pain and eliminates the need for incision care. Notably, although the EBD group experienced a faster recovery, there was a non-significant difference in the “time to first meal” between the two groups (*P* = 0.305), likely because both procedures had minimal impact on gastrointestinal function.

The overall complication rate in the CED group was 25.81%, significantly higher than the 3.57% observed in the EBD group (*P* = 0.044). Complications in the CED group were primarily due to drain-related issues, including dislodgement (9.68%) and obstruction (3.23%), followed by incision infections (6.45%) and abdominal adhesions (3.23%). These complications are closely associated with the invasive nature of CED surgery, which requires the placement of an external drain (T-tube or catheter) in the gallbladder or cyst. Children with thinner abdominal walls and greater mobility are more susceptible to drain displacement due to positional changes or external pulling (7). Additionally, the abdominal wall incision from open or laparoscopic surgery compromises skin barrier function, while intraoperative electrocoagulation or suture techniques may cause local ischemia and necrosis, creating an environment conducive to bacterial colonization (24). In the CED group, one child developed postoperative intestinal adhesions, and intraoperative findings revealed extensive fibrosis of the hepatic-duodenal ligament, consistent with the “secondary surgery adhesion risk model,” which suggests that more traumatic initial surgeries increase the likelihood of adhesions in subsequent radical procedures (25).

In contrast, EBD offers greater stability by utilizing the endoscopic placement of a nasobiliary tube or stent, eliminating the need for an abdominal wall incision. The drain is secured through the nasal cavity or intestine, reducing the risk of dislodgement or obstruction. In this study, no drain dislodgement or obstruction occurred in the EBD group, and only one case of transient hyperamylasemia was observed, potentially related to the temporary elevation of pancreatic duct pressure during ERCP (26). This finding aligns with the report by Tsuchiya et al., which noted only one case of mild pancreatitis after EBD in 15 children with CC (8), indicating that the safety of ERCP in children can be further improved through technical advancements.

Compared to the CED group, the interval to definitive surgery was shorter in the EBD group (median: 14 days vs. 21 days, *P* = 0.002), with a lower rate of intermediate conversion to open surgery (3.57% vs. 29.03%, *P* = 0.024), and lower levels of TB (8.40 vs. 16.20 μmol/L, *P* = 0.019) and transaminases (ALT: 36 vs. 49 U/L; AST: 37 vs. 49 U/L, *P* < 0.01). These findings suggest that EBD can enhance preoperative preparation for radical resection and improve the success rate of minimally invasive procedures. The shortened interval reflects the superior efficacy of EBD in relieving cholestasis and systemic inflammation while reducing pericatheteric edema and fibrosis—key factors for the safe resection of cysts. Although CED provides direct bile drainage for immediate symptomatic relief and inflammation control, its invasive nature may exacerbate systemic inflammatory responses and delay hepatic function recovery (27). Similarly, Sun et al. (16) demonstrated that effective biliary decompression through endoscopic stenting can shorten the inflammatory “cooling” period, allowing for earlier definitive intervention. In contrast, Ten Broek et al. noted that the significantly higher conversion rates to open surgery in the CED group may be attributed to CED-induced peritoneal adhesions and fibrosis, which distort hepatobiliary anatomy and complicate subsequent minimally invasive resections. The rapid biochemical improvement with EBD is consistent with Tsuchiya et al. (8), who suggest that accelerated recovery is due to reduced medical trauma and preservation of biliary anatomy. However, technical limitations (mismatched instrumentation for younger patients’ biliary tracts) may temporarily hinder the broader application of EBD (17). Overall, these findings support using EBD as a physiologically favorable transitional strategy to expedite radical surgery, reduce perioperative risks, and improve the success rate of minimally invasive procedures.

The EBD, through internal stents or nasobiliary drainage, not only ensures effective bile drainage but also preserves the continuity of the biliary physiological channels. Additionally, intraoperative cholangiography during EBD provides a clear visualization of the pancreaticobiliary duct and allows for detecting abnormalities in pancreaticobiliary flow (32 cases of PBM were identified in this study). Compared to magnetic resonance cholangiopancreatography, ERCP offers the advantage of identifying abnormalities in small bile ducts and paracolic hepatic ducts, which is crucial for planning subsequent radical surgeries, such as choledochal cyst resection and hepatic duct jejunostomy (13). In this research, the interval between second-stage radical surgeries was significantly shorter in the EBD group than in the CED group, further highlighting the role of EBD in controlling inflammation and optimizing surgical timing.

This study has several limitations: (1) Despite using strict inclusion and exclusion criteria, retrospective studies inherently face challenges in controlling for confounding factors (variations in the surgical team experience and nutritional status of the children). For instance, the EBD group was treated by three surgeons with specialized endoscopic training, whereas the CED group may have had different surgeons, potentially influencing the outcomes. (2) The relatively small sample size of 59 cases and the lack of long-term follow-up data (incidence of cholangiocarcinoma, risk of anastomotic stenosis) limit the ability to assess the long-term impact of EBD. Future prospective multicenter randomized controlled trials with larger sample sizes and extended follow-up are needed to verify the efficacy and safety of EBD. Moreover, this study was conducted in Wuhan Children’s Hospital (a specialized pediatric ERCP center in China), whereas most primary hospitals still rely on CED techniques. Establishing a standardized training system for EBD is, therefore, essential. This study used adult-standard equipment and accessories, highlighting the need to develop finer-diameter biliary stents and pediatric-specific endoscopic tools to accommodate better the unique anatomical characteristics of children's digestive and biliary tracts.

EBD has indicated significant advantages in treating CC complicated by severe cholangitis in children. Its minimally invasive nature, safety, efficiency, and low complication rate make it a highly favorable alternative to traditional external drainage. Moving forward, the standardization and broader adoption of ERCP in pediatric care should be protected through technological advancements and multidisciplinary collaboration to improve clinical outcomes for this condition further.

## Data Availability

The datasets presented in this study can be found in online repositories. The names of the repository/repositories and accession number(s) can be found in the article/Supplementary Material.
